# A roadmap to a cure

**DOI:** 10.1186/1742-4690-9-S1-I11

**Published:** 2012-05-25

**Authors:** Mario Stevenson

**Affiliations:** 1 University of Miami, Leonard M. Miller, Miami, USA

## 

Highly active anti-retroviral therapy (HAART) effects sustained suppression of viral replication in infected individuals. Despite this, viral replication rapidly resumes if therapy is interrupted. The prevailing view is that viral persistence during HAART is sustained by a reservoir of latently infected, quiescent CD4+ T-lymphocytes, a view that is supported by the apparent lack of viral evolution in the resting CD4+ T cell reservoir and the apparent lack of change in low level plasma viremia when therapy is intensified. A less popular view is that viral persistence in HAART may be sustained by a low level of ongoing or “cryptic” replication, a view that is supported in part by our recent work measuring unintegrated cDNA and specifically episomal cDNA in HAART- treated patients.

In order to characterize the reservoirs that persist in the face of HAART, we have been examining the dynamics of HIV-1 in tissue viral reservoirs and the virologic response to therapy in lymphoid tissue. Lymphoid tissue was obtained from patients at various intervals after HAART initiation and the virologic response as well as intracellular drug levels in cells from lymphoid tissue was examined. Our analysis indicates that suppressive therapy is limited in its ability to curtail HIV-1 replication in lymphoid tissue and that this may be a consequence of poor intracellular sequestration of some antiretrovirals in cells of lymphoid tissue. These results have implications for strategies aimed at limiting viral persistence in the face of HAART.

